# Ecotoxicity of soil contaminated with diesel fuel and biodiesel

**DOI:** 10.1038/s41598-020-73469-3

**Published:** 2020-10-02

**Authors:** Małgorzata Hawrot-Paw, Adam Koniuszy, Grzegorz Zając, Joanna Szyszlak-Bargłowicz

**Affiliations:** 1 Department of Renewable Energy Engineering, West Pomeranian University of Technology, Pawla VI 1, 71-459 Szczecin, Poland; 2 Department of Power Engineering and Transportation, Faculty of Production Engineering, University of Life Sciences in Lublin, Gleboka 28, 20-612 Lublin, Poland

**Keywords:** Environmental sciences, Environmental impact

## Abstract

Fuels and their components accumulate in soil, and many soil organisms are exposed to this pollution. Compared to intensive research on the effect of conventional fuel on soil, very few studies have been conducted on soil ecotoxicity of biofuels. Considering the limited information available, the present study evaluated the changes caused by the presence of biodiesel and diesel fuel in soil. The reaction of higher plants and soil organisms (microbial communities and invertebrates) was analysed. Conventional diesel oil and two types of biodiesel (commercial and laboratory-made) were introduced into the soil. Two levels of contamination were applied—5 and 15% (w/w per dry matter of soil). The plate method was used to enumerate microorganisms from soil contaminated with biodiesel and diesel fuel. Phytotoxicity tests were conducted by a 3-day bioassay based on the seed germination and root growth of higher plant species (*Sorghum saccharatum* and *Sinapis alba*). Fourteen-day ecotoxicity tests on earthworm were performed using *Eisenia fetida*. Based on the results of the conducted tests it was found out that the organisms reacted to the presence of fuels in a diverse manner. As to the microorganisms, both the growth and reduction of their number were noted. The reaction depended on the group of microorganisms, type of fuel and dose of contamination. The lipolytic and amylolytic microorganisms as well as *Pseudomonas fluorescens* bacteria were particularly sensitive to the presence of fuels, especially biodiesel. Fuels, even at a high dose, stimulated the growth of fungi. Monocotyledonous sugar sorghum plants were more sensitive to the presence of fuels than dicotyledonous white mustard. There was also a significant negative impact of contamination level on plant growth and development. Biodiesel, to a greater extent than conventional fuel, adversely affected the survival and volume of earthworms.

## Introduction

Energy security and political, economic and, above all, environmental benefits, including counteracting adverse climate changes associated with the use of conventional fuels, are the main reasons for the development of biofuels market^1,2^.

One of the most popular biofuels is biodiesel. The biofuel produced from renewable substrates seems to be an ideal source of energy. Its properties are similar to diesel, while greenhouse gas emissions are lower^3–5^. Combustion of biodiesel is also associated with lower emission of sulfur compounds, aromatic compounds^6^, at increased NO_x_ emissions^7^ or a little decrease compared to diesel at low loads under low and medium engine speeds^8^. The use of biodiesel on an increasingly larger scale, also as a biocomponent for conventional fuels, raises concerns about its impact on the environment resulting not only from the use, but also the production process of biofuel, improper use of equipment or emergency leaks during storage or transport.

Biodiesel can accumulate in the environment and may affect soil and its biodiversity^9,10^. The toxicity of biodiesel has not yet been sufficiently investigated. Comparatively few studies were carried out on the ecotoxicology of biofuels and their results are vary widely. Some studies indicate less or comparable effects induced by biofuels in comparison to fossil fuels^11^. Biodiesel can provide a good source of carbon and energy to various autochthonous soil microorganisms^12,13^ and showed a positive effect on growth and development of some soil microorganisms^14^. However, other available results indicate that biofuel may have an adverse impact on the environment^15^. Effluent from the biodiesel production have negative impact on physicochemical properties of soil and degrade soil quality^16^. Biodiesel contaminated soil exhibited genotoxic and mutagenic effects^17^. Biofuel had a negative influence on the biometric and physiological parameters of plants^18^, reduce seedling germination^19^ and soil microbial biomass^20^. The differences in test results regarding toxicity depends, among other reasons, on the different chemical composition of biofuels^11,21^, levels of contaminants^22^ and the different research methods.

The most sensitive indicator of changes in the environment, used to assess the negative effects caused by environmental contaminants, are biological methods. Bioassays allow not only to detect a toxic substance, but also to quantify its negative impact^23^. In assessing the level of environmental contamination, pollutants are also subjected to chemical analyses, which are, however, relatively expensive. Pollutants are generally a mixture of different compounds, which makes their determination even more difficult.

In contrast to chemical analyses, bioindication methods also detect the presence of metabolites. Bioassays detect adverse effects of single compounds and complex chemical mixtures^24^. In the toxicological evaluation of soil and soil samples, soil microorganisms, earthworms and plants are successfully used. The use of a set of biotests allows a full impact assessment^25^, taking into account synergism or antagonism^26,27^.

The objective of this study was to assess the changes caused by the presence of biodiesel in the soil compare to fossil diesel fuel. We analysed the reaction of soil microbiome based on the number and activity of microorganisms, plants (phytotoxicity test) and invertebrates, earthworms (zootoxicity test). To the best of our knowledge, the present study is the first to analyse the toxic effects of two different types of biodiesel (commercial and laboratory-made) and conventional fuel on biological life in soil by using a battery of ecotoxicological tests, considering different levels of trophic life. We hypothesized that (1) the contamination of soil by biodiesel can alter the biological properties of soil and (2) the toxic effect is dependent on the concentration of biofuel and the type of soil organisms.

## Materials and methods

### Soil

The research was carried out in clayey sand (Table 1). The material was taken from the depth of 0–15 cm of the arable topsoil in the Agricultural Experimental Station in Lipnik (53°20′ N, 14°58′ E), belonging to the West Pomeranian University of Technology in Szczecin.

**Table 1:** Physiochemical properties of the soil.

Percentage of fraction			C_org_	N	pH_KCl_
2.0–0.05	0.05–0.002	< 0.002	g∙kg^−1^ DM soil		
82.4	16.6	1.2	11	1.1	6.41

### Experimental set-up and analysis

Three types of fuels were used in the tests: diesel oil collected from a distributor at a petrol station (D), biodiesel supplied by one of the Polish producers (BDI) and biodiesel prepared in the laboratory in the transesterification process with rape oil and methanol using KOH (BDII). As part of the analyses, the viscosity of fuels was also determined using the Brookfield DV-II + viscometer.

After being brought to the laboratory, the soil samples were air-dried, passed through a sieve of 2-mm mesh, and their actual moisture level was determined using the drying-weight method. On this basis, the soil was brought to 60% of the total water capacity and this humidity was maintained during the experiment. Fuels were introduced into the soil in a dose of 5 and 15% (w/w to DM soil). The soil surface was dosed with diesel fuel and biodiesel by the sprinkling method. After 10 min, the soil samples were thoroughly mixed. One object was left uncontaminated as a control (K). Soil samples weighing 2 kg were placed in polyethylene containers and incubated at 22 ± 1 °C for 28 days. As part of the study, after 4 weeks of the experiment, microbiological tests as well as phytotoxicity and zootoxicity tests were performed. Soil samples for the analysis were collected from each container. All the estimations for particular bioassays were performed in three replicates.

Microbiological analyses included the determination of basic taxonomic groups of soil microorganisms (bacteria, actinomycetes, fungi), as well as proteolytic, amylolytic, lipolytic and cellulolytic microorganisms, bacteria of the genus *Azotobacter* and bacteria *Pseudomonas fluorescens*. The determinations were carried out by the soil culture dilution method using culture media appropriate for particular groups of microorganisms (Table 2). The results are presented as colony forming units (CFU) calculated on 1 g of dry matter of soil.

**Table 2:** Specific culture requirements of microorganisms.

Microorganisms	Culture medium	Incubation	
		Temperature (°C)	Time (days)
Bacteria	Bunt and Rovira^28^	20	3
Actinomycetes	Cyganov and Žukov^29^	20	7
Fungi	agar medium, Martin^30^	20	5
Proteolytic	medium with milk, Kędzia and Konar^31^	20	3
Amylolytic	medium with starch, Cooney and Emerson^32^	20	3
Lipolytic	medium with tributyrin, Burbianka and Pliszka^33^	20	3
Cellulolytic	Maliszewska^34^	20	7
*Azotobacter*	Fenglerowa^35^	20	7
*Pseudomonas fluorescens*	King B medium	26	3

By comparing the number of bacteria and actinomycetes to the number of fungi, the so-called SR fertility coefficient was determined^36^, the higher values of which are more favorable^37^.

Acute toxicity microbiotests Phytotoxkit F were carried out using with monocotyl and dicotyl plant species, sorghum sugar (*Sorghum saccharatum*) and white mustard (*Sinapis alba*). The germination process and inhibition of plant root growth were evaluated on test plates after 3 days of incubation in the dark at 25 °C, relative to the control soil.

The test with the use of earthworms was carried out in glass containers in each of which 0.5 kg of contaminated soil was placed. The soil samples, after 28 days of incubation with fuels, were pre-mixed with 5 g of ground rye straw providing a source of food. The containers were stored in the dark at 22 ± 1 °C, maintaining a constant humidity of 60% maximum water holding capacity. After 14 days, the number of alive earthworms and their mass were determined. Based on them, mortality (in %) and weight loss (in %) were calculated in relation to the initial values.

### Statistical analysis

All the statistical analyses were carried out using the statistical software package for Windows (Statistica version 13.3; Dell Inc., Tulsa, OK, USA). Analysis of variance and post hoc Newman–Keuls tests were performed at statistical significance of *P* ≤ 0.05.

## Discussion of results

**Figure 1:**
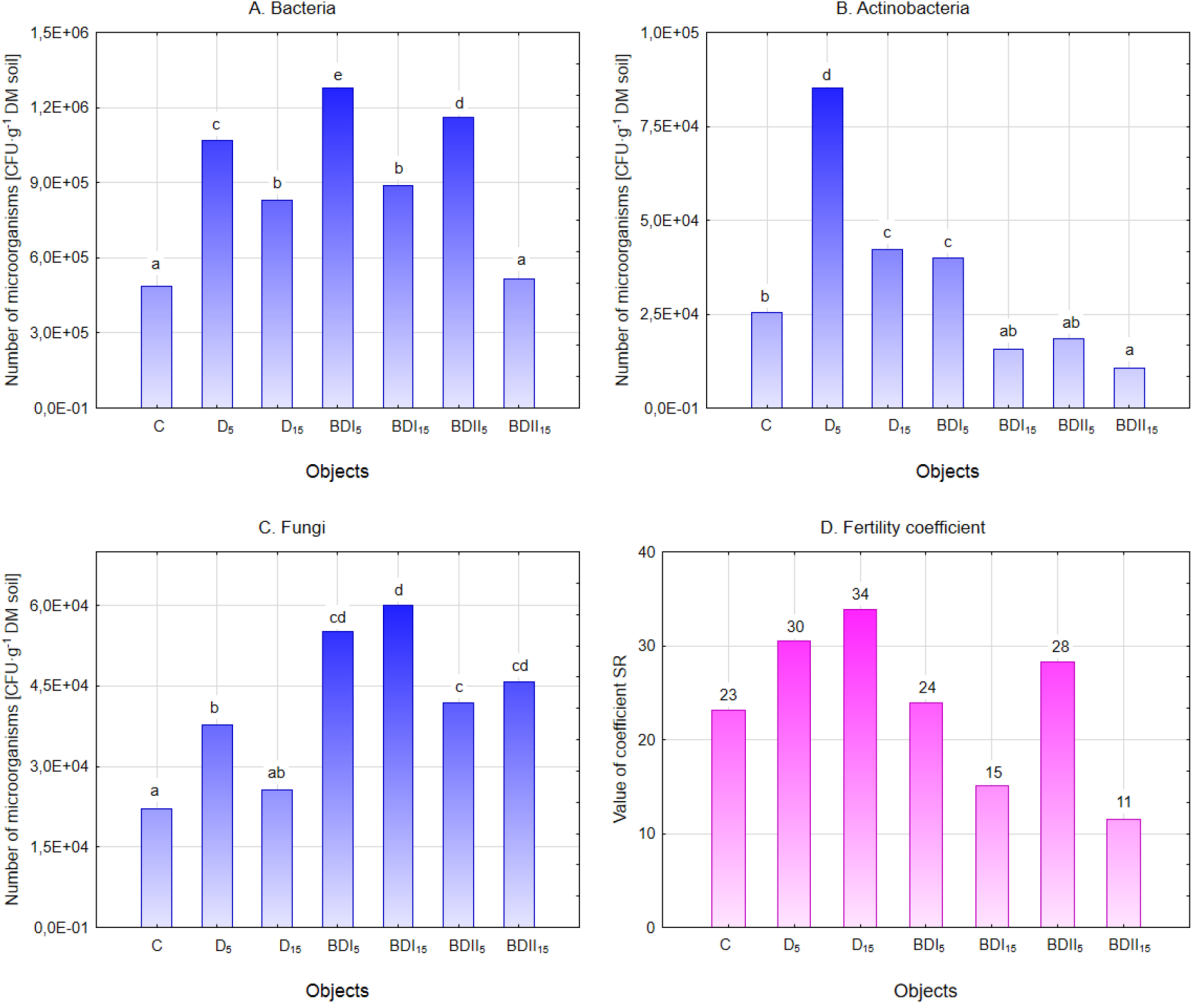
The number of basic taxonomic groups of microorganisms (mean over each columns not marked with the same letter is significantly different at *P* ≤ 0.05) and the fertility coefficient in fuel contaminated soil.

Contamination can affect soil ecology, including its microbial abundance and diversity^38^. The impact of diesel oil and biofuels on the number and activity of soil microorganisms is not conclusive^39^. Fuels introduced into the soil caused an increase in the number of bacteria (Fig. 1A). In general, the number of microorganisms in soil contaminated with petroleum derivatives changes over time^40^. Previous studies have observed a positive effect of diesel fuel on bacteria^40,41^, during the first weeks after contamination. Hazim and Al-Ani^42^ analysed the effect of petroleum hydrocarbon contamination at 5% and 10% concentrations on soil microorganisms. The authors observed a significant decrease in the number of heterotrophic bacteria and an inverse correlation between the concentration of the contaminant and the count of these species. The largest changes were recorded in the BDI_5_—1.28 × 10^6^ CFU g^−1^ DM soil. In all the tests, the number of bacteria decreased significantly with the increase in the concentration of the contaminant to 15%.

The negative effect of increased concentration of contaminant was also observed on actinomycetes (Fig. 1B). These microorganisms were less sensitive to the presence of conventional fuel, while particularly adverse changes were noted in the soil contaminated with biodiesel prepared in the laboratory. The values determined in the BDII_5_ and BDII_15_ were by 28% and 58% lower compared to the unpolluted soil. Considering the participation of actinomycetes in the processes of organic metabolism in the soil, the observed changes were not favourable.

The presence of fuels in the soil stimulated the growth and development of fungi (Fig. 1C). Molina-Barahona et al.^43^ also received similar results with regard to diesel oil in the studies taking into account the effect of biodiesel on soil microbiota. In the control sample, the average number of fungi was 2.21 × 10^4^ CFU g^−1^ DM soil, while under the influence of diesel fuel it increased in the range of 71–16%, in D_5_ and D_15_, respectively. In the study carried out by Hazim and Al-Ani^42^, soil contamination with 5% and 10% diesel oil increased fungi count by 73% and 139%, respectively. This finding demonstrated the capacity of fungi to use diesel hydrocarbons as an energy source and their potential ability to biodegrade diesel oil^44^. Although mycoremediation is not yet well understood and is currently being researched, the use of fungi for the biodegradation of petroleum-based hydrocarbons is more advantageous than the use of bacteria^45^. Fungi can transform most recalcitrant fuel components, including high-molecular-weight polycyclic aromatic hydrocarbons^46^, under extreme environmental conditions that are intolerable for most bacteria^47^. Even higher values were recorded in the presence of biodiesel, regardless of its origin. In this case, the increase in the dose of contamination had a stimulating effect on the number of fungi.

The constituents of pollutants introduced into the soil, as well as products that arise during their decomposition, may have an adverse effect on microorganisms, and hence on the quality and fertility of the soil related to their activity^40^. In the presence of diesel, the SR value increased by more than 30% in the D_5_ sample and almost 50% in the D_15_ compared to the control (Fig. 1D). The reduction in SR coefficient is less favorable and shows a stronger development of fungi. Such a situation was recorded in the presence of a higher dose of commercial biodiesel (BDI_15_) and in the BDII_15_ sample, where the value of fertility coefficient in relation to the control values was by 50% lower. Biodeterioration studies on biodiesel indicate that biofuel is more susceptible to microbial contamination than petroleum fuels^48^, and the components of biodiesel are very susceptible to degradation by fungi^49^.

**Figure 2:**
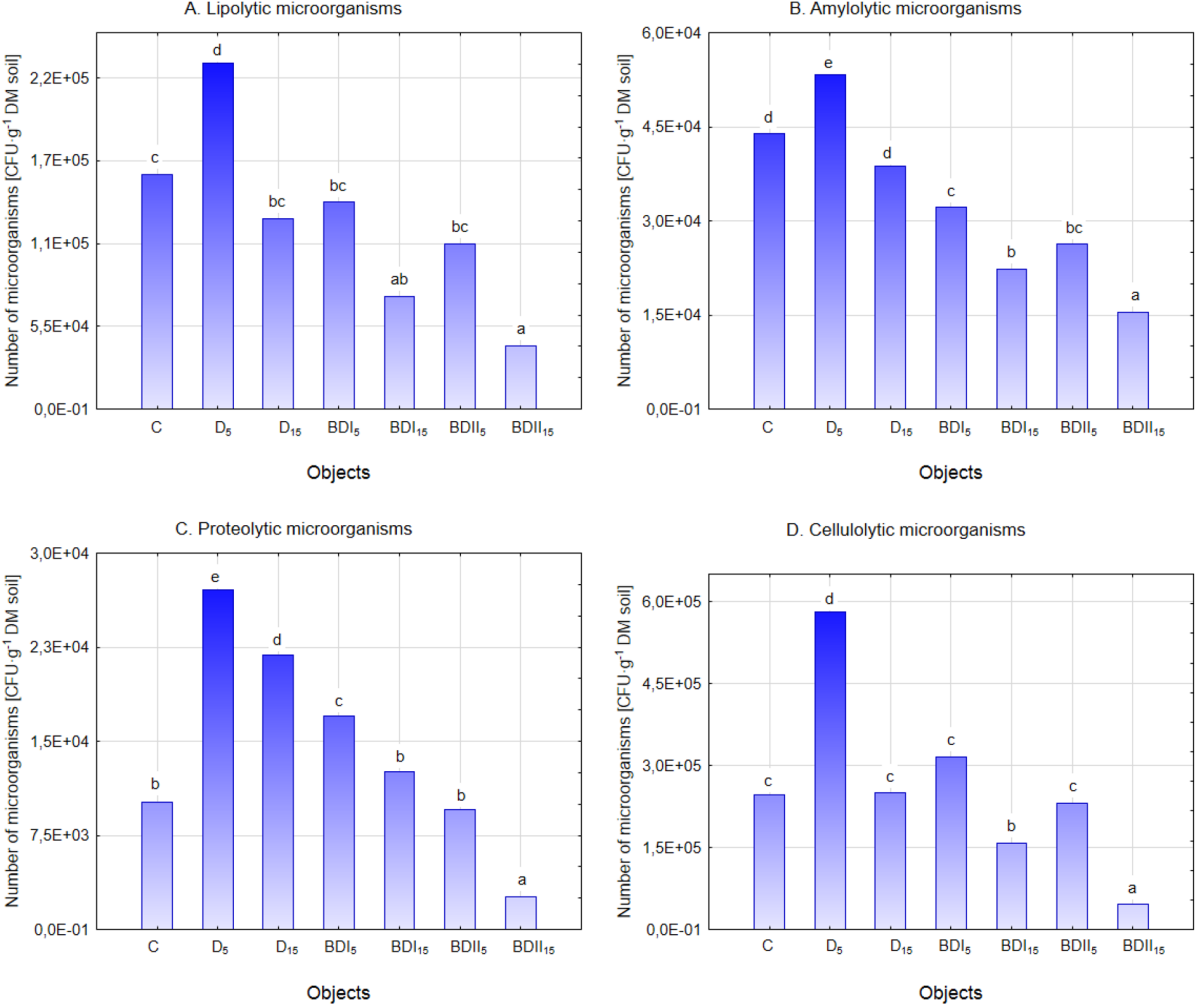
The number of selected metabolic groups of microorganisms in fuel contaminated soil (mean over each columns not marked with the same letter is significantly different at *P* ≤ 0.05).

The stimulating influence of diesel oil in the concentration of 5% on the number of all metabolic groups of soil microorganisms (Fig. 2A–D) was noted, while in the 15% dose only in relation to proteolytic microorganisms. A different reaction was observed in soil contaminated with biofuels. Fatty acid esters, which are included in the biodiesel, are synthesized also in the natural environment^50^ and should not adversely affect microorganisms, however, for lipolytic and amylolytic organisms the presence of biofuel regardless of its origin and dose, limited the number in comparison to control soil (C) and to the samples contaminated with conventional fuel (D_5_ and D_15_). The negative impact of biofuels on the number of metabolic soil microbiocenosis groups important from the point of view of the proper functioning of the soil environment was also observed in earlier studies. It should be noted that apart from the type of contamination and its dose, the type of soil^51^ has a significant influence on the observed changes, especially on the content of colloidal fractions.

Biodiesel II at a dose of 15% reduced the number of proteolytic microorganisms. After 28 days of experiment, only 2.61 × 10^3^ CFU g^−1^ DM soil was determined, and the reduction of their number in soil may adversely affect the mineralization processes of organic nitrogen compounds. Biodiesel I and II at the 15% dose also had a negative effect on cellulolytic microorganisms that participate in the carbon cycle^52^. As in the case of all the studied metabolic groups, the smallest number was recorded in the BDII_15_ (4.61 × 10^3^ CFU g^−1^ DM soil).

**Figure 3:**
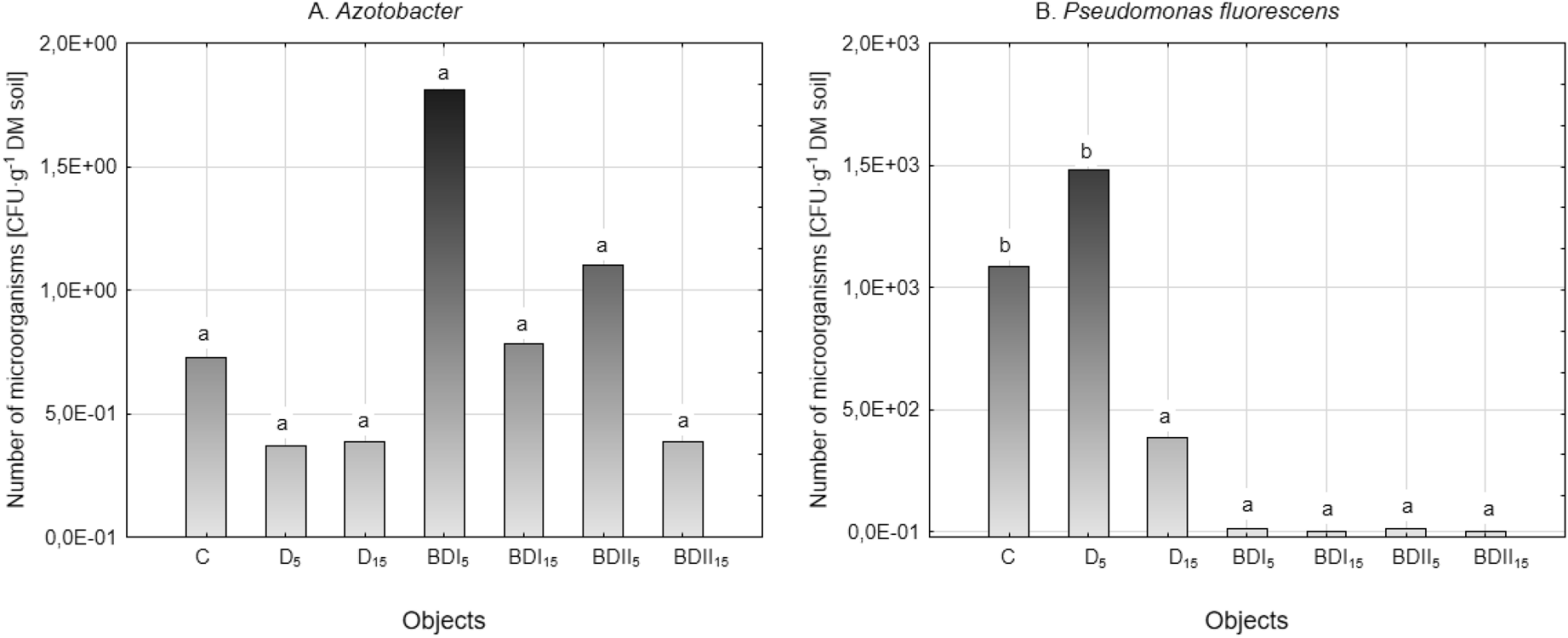
The number of *Azotobacter* and *Pseudomonas fluorescens* in fuel contaminated soil (mean over each columns not marked with the same letter is significantly different at *P* ≤ 0.05).

Fuels introduced into the soil stimulated or reduced the number of bacteria of the genus *Azotobacter* (Fig. 3A), depending on the type of contamination and its dose, but these were not statistically significant differences. The presence of *Pseudomonas fluorescens* in the soil is important due to the substances produced by these microorganisms that inhibit the growth and development of plant pathogens^53,54^. van Dorst et al.^55^ found that the toxicity of diesel fuel reduced biodiversity and that the new community was dominated by only a few microbial species, mainly *Pseudomonas*. Only in the sample D_5_ the number of these bacteria was higher compared to the control (1.43 × 10^3^ CFU g^−1^ DM soil). In soil contaminated with diesel oil at a dose of 15% and biofuels at a dose of 5% and 15%, regardless of their origin (BDI and BDII), a significant reduction in the number of *Pseudomonas fluorescens* in the range from 65 to 100% was noted (Fig. 3B). The results indicate that diesel fuel toxicity leads to a clear selection of microorganisms and the displacement of intolerable strains by other groups of tolerable microorganisms. Perhaps, this is because biofuel components are not a good source of carbon and energy for these microorganisms, even though various studies show the biodegradation potential of some *Pseudomonas* species^56,57^. Microbial populations can be reduced and even eliminated in the presence of fuel components. Some microorganisms require time to adapt to a contaminated environment^40^.

A different reaction of microorganisms to the presence of individual fuels may result from their different chemical composition^48^. Biodiesel is mainly a mixture of fatty acid methyl esters^58^. Diesel fuels consist of the hydrocarbon groups, including n-paraffins, isoparaffins, naphthenes, olefins and aromatics^59^. In soils, aromatic hydrocarbons have high durability and varied toxicity^60^. Biodiesel is different from petroleum fuels, and the properties of the two types of biodiesel used in this study are also different. Unlike the biofuel produced in laboratory, commercial biodiesel contains additives for controlling microbial contamination^61^. In addition, there were also differences in the physical properties of fuels, such as viscosity. At 20 °C, the lowest values (4.534 mm^2^ s^−1^) were determined for diesel fuel, and the highest for biodiesel produced in the laboratory (7.744 mm^2^ s^−1^). The kinematic viscosity of commercial biodiesel was 6.813 mm^2^ s^−1^ . Higher viscosity fuels more slowly penetrate and spread in soil^62^, which also affects their availability for microorganisms.

Some microorganisms from diesel-contaminated soils can be used as an effective candidate for bioremediation process^63,64^. For others petroleum fuels are toxic, which may be the effect of direct adverse effect on cells or indirectly from changes caused in the soil environment. The properties of the soil, its reaction or texture, can affect organisms^26^. Biodiesel is relatively resistant to the activity of natural microflora and is decomposed more slowly in a complex environment such as soil^65^; hence, its effect on the environment may persist over a longer period of time.

**Figure 4:**
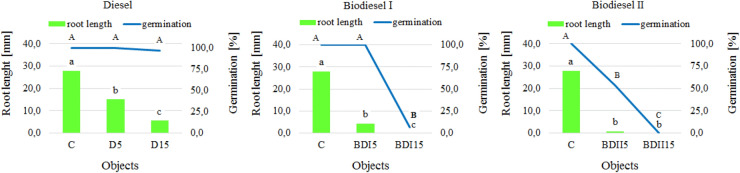
Average root length and percentage of *Sorghum saccharatum* germinating seeds depending on the type of contamination and dose (mean over each columns not marked with the same letter is significantly different at *P* ≤ 0.05).

**Figure 5:**
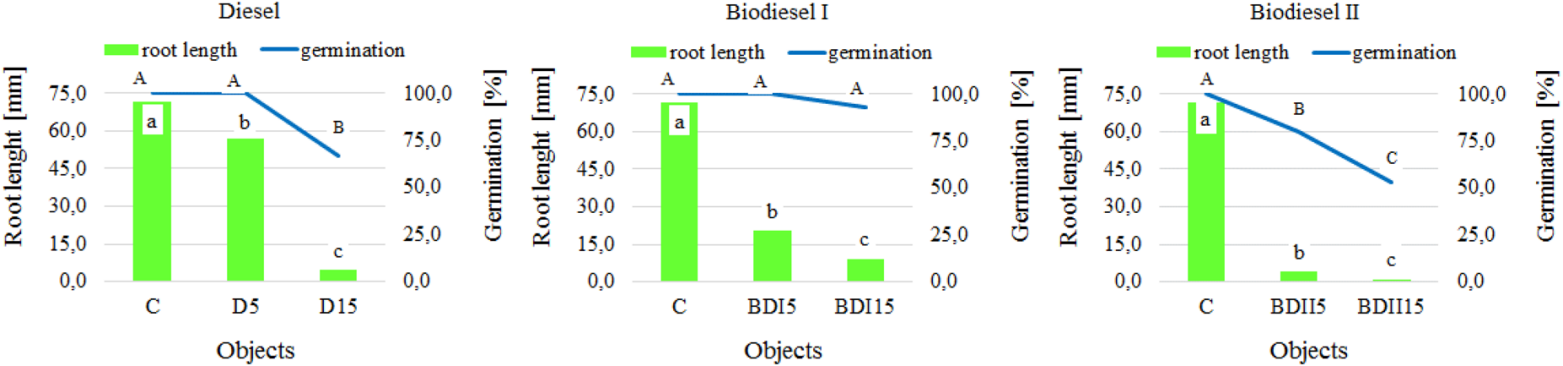
Average root length and percentage of *Sinapsis alba* germinating seeds depending on the type of contamination and dose (mean over each columns not marked with the same letter is significantly different at *P* ≤ 0.05).

The presence of diesel oil and biofuels in the soil had a negative impact on the germination, growth and development of both the sugar sorghum (Fig. 4) and white mustard (Fig. 5). Sprouting and root growth are the two critical stages of plant development particularly sensitive to environmental pollution^66^. The root length of the studied plants significantly decreased with the increase of the level of contamination, regardless of the type of fuel, with the highest inhibition observed in the samples contaminated with biodiesel produced in the laboratory. Some studies confirmed that biodiesel, same as diesel fuel, has a phytotoxic potential^18,67^. According to Bamgbose and Anderson^68^ biodiesel toxicity was more evident at high concentrations.

**Figure 6:**
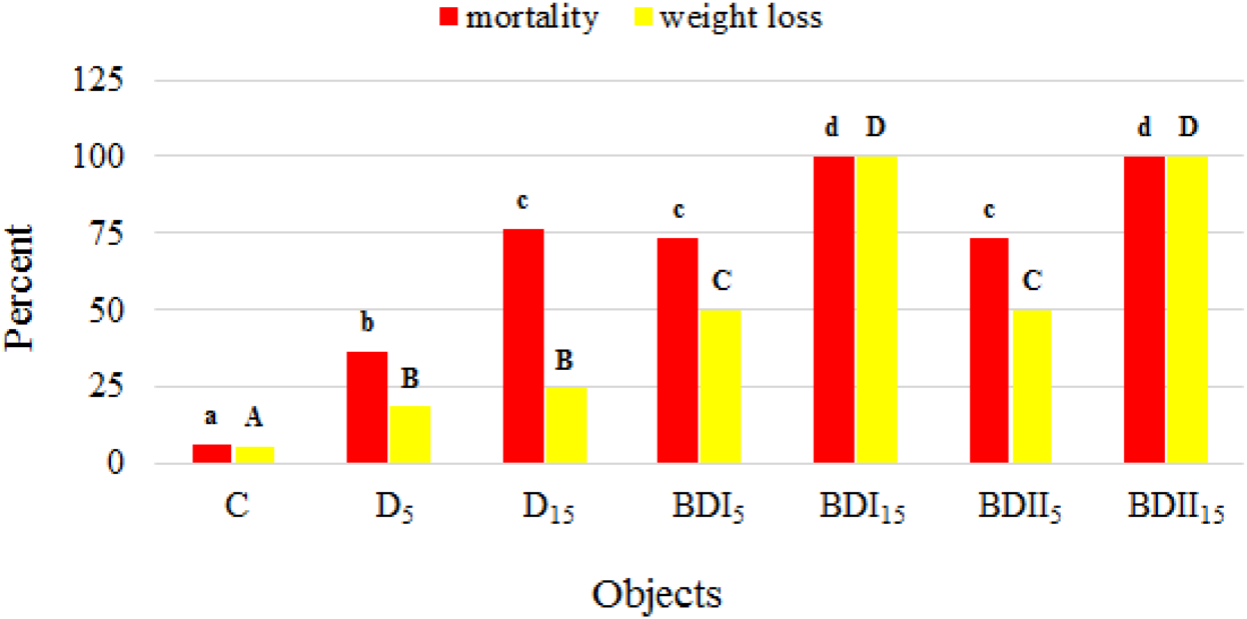
The earthworms mortality and weight loss after incubation in fuel contaminated soil (mean over each columns not marked with the same letter is significantly different at *P* ≤ 0.05).

Earthworms are an important element of the soil environment, they participate in the processes of soil formation, the circulation of organic matter and affect the physical and chemical properties of soil^69^. During the introduction of earthworms into the containers, the escape reaction was observed at the first contact with the soil contaminated with diesel oil, hence the containers were secured with gauze. There were no such behaviours in the soil sample contaminated with biofuels, and the earthworms quickly penetrated the soil. Before starting the experiment the soil was provided with food for the earthworms, also, the appropriate humidity and temperature were maintained, however at the end of the experiment, the number of test organisms in the particular experimental samples decreased in the range from 7 to 100% (Fig. 6). Particularly unfavourable conditions for their development were observed in the soil contaminated with biodiesel I and II at a dose of 15%. The fuels also had a negative impact on the weight of earthworms. The value of this parameter decreased after 14 days of the biotest in the range from approx. 19% in the D_5_ sample to approx. 50% in the BDI_5_ and BDII_5_. In the study carried out by Bamgbose and Anderson^70^ plant-based biodiesel were less toxic for earthworm in 14-day mortality test compare to diesel fuel however, exposition on this pollutant caused more weight loss, coiling, posterior and anterior fragmentation, and excessive discharge of coelomic fluid.

Bioassays are a very useful tool for monitoring environmental pollution, especially for complex pollutants such as fuels. Similar to conventional fuel, biodiesel and its effluent can contribute to the deterioration of physical and chemical parameters of soil^71^, which has a negative effect on soil biology and function. The influence of biofuels on microorganisms, plants and animals was generally less favourable than the impact of conventional fuels. The reaction of the studied groups of organisms to the type of biofuel was also different. To biodiesel, at the production stage, among others there are introduced depressors, antioxidants that may adversely affect biotic elements of the environment^72^, however, this biofuel prepared in the laboratory, lacking a number of these additives, showed more toxic properties. In the soil, methanol toxic to organisms^3^ could appear due to a reversal of the transesterification process.

## Conclusions

The influence of fuels on the studied groups of organisms was ambiguous. Not only the type of fuel, but also the level of contamination was important. The organisms showed different sensitivity to the presence of individual pollutants, however, for the most part, the biofuels caused more adverse changes. The toxicity of the fuels used in the tests can be ordered in the following way: BII > BI > D. The use of soil-specific organisms in the tests has allowed to assess the impact of pollution on the biodiversity of the ecosystem and its quality.

The negative impact of fuels on microorganisms may disturb the proper functioning of the soil environment. Fuels decrease or increase the growth of various microorganisms, depending on their concentration and types. Soil pollution with diesel oil had a positive effect on fungal population, which implies that its components could be used as a source of carbon and energy. The proliferation of fungi was stimulated by biofuels even at high concentrations of the contaminant. This indicates the ability of fungi to degrade or transform toxic fuel components and reduce their negative effect on the biological properties of soil. Contaminated soil limits the possibility of plant growth and development. It also negatively affects the participating in the soil formation processes, responsible for shaping its structure and for the redistribution of organic matter.

The results of ecotoxicity tests indicate that some organisms can be used for biological remediation of soils contaminated with fuels, including biodiesel. The sensitivity of some tested organisms to the presence of biofuel, which depends on the concentration of the contaminant, indicates the possibility of using them as potential indicators of biodiesel toxicity in soils and for assessing the efficacy of the biodegradation/phytoremediation process. Additional studies are required to determine the toxicity effect of biodiesel as a function of time (long-term effect) and to conduct detailed analysis of the physicochemical properties of soils after contamination with fuels.
